# Investigation of* Anaplasma marginale* Seroprevalence in a Traditionally Managed Large California Beef Herd

**DOI:** 10.1155/2016/6186078

**Published:** 2016-08-28

**Authors:** Thomas R. Tucker, Sharif S. Aly, John Maas, Josh S. Davy, Janet E. Foley

**Affiliations:** ^1^Departments of Medicine and Epidemiology, School of Veterinary Medicine, University of California, Davis, Davis, CA 95616, USA; ^2^Department of Population Health and Reproduction, School of Veterinary Medicine, University of California, Davis, Davis, CA 95616, USA; ^3^Veterinary Medicine Teaching and Research Center, School of Veterinary Medicine, University of California, Davis, Tulare, CA 95616, USA; ^4^Department of Population Health and Reproduction, University of California, Davis, Davis, CA 95616, USA; ^5^Division of Agriculture and Natural Resources, University of California, Davis, Red Bluff, CA 96080, USA

## Abstract

Recent observations by stakeholders suggested that ecosystem changes may be driving an increased incidence of bovine erythrocytic anaplasmosis, resulting in a reemerging cattle disease in California. The objective of this prospective cohort study was to estimate the incidence of* Anaplasma marginale* infection using seroconversion in a northern California beef cattle herd. A total of 143 Black Angus cattle (106 prebreeding heifers and 37 cows) were enrolled in the study. Serum samples were collected to determine* Anaplasma marginale* seroprevalence using a commercially available competitive enzyme-linked immunosorbent assay test kit. Repeat sampling was performed in seronegative animals to determine the incidence density rate from March through September (2013). Seroprevalence of heifers was significantly lower than that of cows at the beginning of the study (*P* < 0.001) but not at study completion (*P* = 0.075). Incidence density rate of* Anaplasma marginale* infection was 8.17 (95% confidence interval: 6.04, 10.81) cases per 1000 cow-days during the study period. Study cattle became* Anaplasma marginale* seropositive and likely carriers protected from severe clinical disease that might have occurred had they been first infected as mature adults. No evidence was found within this herd to suggest increased risk for clinical bovine erythrocytic anaplasmosis.

## 1. Introduction

Bovine erythrocytic anaplasmosis, caused by* Anaplasma marginale*, creates millions of dollars of annual losses in California alone [1]. Such economic loss makes anaplasmosis prevention a critical component of herd health management for commercial beef cattle producers. All cattle are susceptible to* A. marginale* infection but development of clinical disease is dependent on age at the time of infection as well as factors such as cattle breed, strain virulence, and vector abundance and other factors affecting animal health such as husbandry [2]. When young animals (<6 months of age) are infected, they generally do not develop clinical disease [3]. In contrast, when mature animals are infected, severe clinical disease may occur, with case-fatality rates reaching 49% in cattle infected after two years of age [4, 5]. Animals surviving infection develop lifelong carrier status and freedom from subsequent clinical anaplasmosis [3, 4, 6].

Bovine anaplasmosis is an endemic vector-borne disease in many parts of California, with tick vectors and both sylvatic (various deer spp.) and livestock reservoirs creating a high risk of infection for naive grazing cattle [7]. Beef cattle managers often move herds of young cattle (<2 years old) to specific pastures throughout the state where high infection prevalence in ticks is thought to occur. The intentional exposure of young cattle to ticks is intended to increase the probability that animals acquire the infection while they are young and attempt to decrease incidence of severe disease when older animals are infected [8]. In California, this management practice should result in high prevalence of young animals with lifelong* A. marginale* carrier status, protected from clinical anaplasmosis [5]. However, over the last three years, ranchers have reported an increased incidence of clinical anaplasmosis which is assumed to be due to a failure of this prophylaxis and high prevalence of* A. marginale *seronegative adult cattle [9]. Ecosystem changes in California have pushed black-tailed deer (*Odocoileus hemionus columbianus*) and mule deer (*Odocoileus hemionus*) from the foothills and into the valleys where they are safer from predation, moving the primary sylvatic reservoir for* A. marginale* to a new region and possibly changing the dynamics of natural exposure to ticks and prevention strategies for clinical anaplasmosis. If true, this would pose a critical herd health challenge of reemergent disease that cattle ranchers and veterinarians must address through new strategic anaplasmosis prophylaxis.

In addition to tick biologic vectors, mechanical vectoring is possible with veterinarians and animal husbandry workers [3, 10]. The organism can be transmitted on drops of blood transferred between animals during vaccination, castration, dehorning, and other routine management practices. Biting flies can also transmit this pathogen if they are interrupted during a blood meal and move from an infected to a naive animal [5, 11–13].

To our knowledge, there are no published studies that report on the true incidence of* A. marginale* infection in California beef cattle herds that are intentionally moved to select pastures for exposure to ticks and prevention of clinical anaplasmosis. The objective of this prospective cohort study was to evaluate the risk of infection in heifers and mature cattle in a large commercial beef cattle herd in California using pasture-based natural infection to prevent clinical anaplasmosis. Comparing seroprevalence between heifers and mature cattle will establish a historic baseline for evaluating efficacy of the natural infection strategy within a herd by determining how many mature cattle were already* A. marginale *carriers and thus resistant to severe disease. We hypothesized that the incidence density rate (IDR) in cows and heifers would be low and would not differ between age classes, supporting claims that adult herds contain high proportions of susceptible animals (>40% [7]* A. marginale* seronegative). Results of this study provide regional insight into the epidemiology of anaplasmosis in California beef cattle and suggest evidence-based recommendations for anaplasmosis prevention in comparable management systems.

## 2. Materials and Methods

### 2.1. Study Population

The study period was from March through September of 2013. A total of 143 animals, part of a commercial herd of Black Angus beef cattle, were enrolled in the study (Table 1). The overall herd had a mean size of 2000 cow/calf pairs and included replacement heifers to maintain a steady population with an approximately 10% cull rate in cows. The home ranch for this herd was in Tehama County, California; however, ranch managers move cattle throughout the state at different times of the year. Cattle were wintered on annual rangeland in Tehama County (6,535 hectares). Summer feed consisted of valley irrigated pasture and mountain meadow irrigated pasture in Tehama County (100 hectares) and Plumas County (975 hectares). The overall herd contained multiple smaller groups of varying numbers. Calves were born on irrigated pastures during midsummer and replacement heifers were selected in the fall. Ranchers managed the herd cattle based on stage of production and pasture carrying capacity by moving animals between groups as necessary for effective management and did not use any anaplasmosis vaccines. Ranchers treated cattle with ivermectin in the fall and doramectin in the spring for internal and external parasites but did not use an acaricide in the herd health program.

Three cohorts were selected for participation based on the likelihood of subsequent accessibility for sample collection and to ensure study events would not interfere with overall herd management. The first cohort (group 2) contained 66 heifers ranging from 15 to 17 months of age at the first sampling date (day 0) and were tested for anaplasmosis from the time they were first placed with the bulls until calving and again two additional times: day 70, just prior to being moved to a second pasture location, and day 193. The ranch manager stated that all of the sites used during this study were thought to contain ticks that were capable of infecting cattle with* A. marginale*. Two additional cohorts (one cohort of heifers and one cohort of cows) were enrolled as they became available to increase sample size and to enable comparison between heifers and mature cows. The two additional cohorts contained 40 heifers (group 1) and 37 cows, respectively, and were enrolled on day 77, at which point they were sampled for anaplasmosis testing and shipped to a second pasture location, where they remained until their final sample collection on day 157. The additional heifer cohort (group 1) ranged from 9 to 12 months of age at day 0. The cows were all >2 years old on day 0. The addition of the second heifer cohort (group 1) and cows allowed comparison of two pasture locations for incidence of anaplasmosis and contrasted the* A. marginale* seroprevalence between cows and heifers.

### 2.2. Specimen Collection

Blood samples were collected by tail vein venipuncture. Approximately 8 mL of whole blood was collected into serum separator tubes and allowed to sit at ambient temperature until clotted. Samples were then centrifuged and approximately 2 mL of serum was transferred into 2.5 mL cryotubes. Cryotubes were stored on ice packs in an insulated container until the end of the day and subsequently held at −20°C for storage until testing. Animals that were seropositive for* A. marginale* were considered carriers and were not tested again. Each animal was inspected for ticks while being processed through a squeeze chute for blood collection.

### 2.3. Sample Analysis

Frozen serum samples were thawed and analyzed for the presence of antibodies to* A. marginale* using a commercially produced competitive enzyme-linked immunosorbent assay (cELISA; VMRD, Pullman, Washington). In previous test validation studies, this cELISA had 95% sensitivity and 98% specificity for diagnosis of* A. marginale* infection in cattle raised within* A. marginale* endemic regions [14]. All laboratory testing was performed according to manufacturer instructions. Three positive and two negative controls were included with every 96-well test plate, as per the manufacturer's instructions. Results were determined using a microplate absorbance spectrophotometer with an optical density wavelength of 630 nm. Serological status was determined using percent inhibition cutoffs provided with the test kit (<30% inhibition read as negative and ≥30% inhibition read as positive) for* A. marginale*.

### 2.4. Statistical Analysis

True prevalence (TP) of anaplasmosis was determined from the measured* A. marginale *seroprevalence using the equation described by Rogan and Gladen [15] using the following formula:

(1) $$\begin{array}{c} TP=\frac{AP+Sp-1}{Se+Sp-1}, \end{array}$$

where apparent prevalence (AP) was calculated from the serologic results of this study and sensitivity (Se) and specificity (Sp) estimates were those reported by the manufacturer. A 95% confidence interval (CI) for the TP, taking into account diagnostic test Se and Sp studies [14] and the number of animals used in those studies, was approximated using the Reiczigel method (Reiczigel online calculator, http://www2.univet.hu/users/jreiczig/prevalence-with-se-sp.html) [16, 17]. True prevalence was reported except where specifically indicated. The TP and AP of anaplasmosis were compared across cohorts using Pearson's chi-square test. The following procedure was used to determine the number of observed* A. marginale* positive animals within each cohort for the chi-square analysis of TP. The TP, previously calculated for each group, was multiplied by the number of animals in the group. The result of the product was rounded to the nearest whole number and used as the estimate of animals that were* A. marginale* seropositive. The TP and AP of anaplasmosis at the initial and final sampling dates within each cohort were compared using McNemar's test (SPSS, Version 22, IBM, Armonk, NY).

Ninety-five percent CIs for IDR were calculated using the method described by Szklo and Nieto [18] with confidence limit factors for Poisson-distributed variables provided by others [19, 20]. The ratio of two IDRs was calculated to estimate the incidence rate ratio (IRR) as described in Szklo and Nieto [18] and their 95% CIs were calculated as proposed by Ederer and Mantel [20]. An approximate chi-square test with 1 degree of freedom was used to conduct hypothesis testing that the IRR was equal to 1 as described by Szklo and Nieto [18].

The study protocol was reviewed and approved by the Institutional Animal Care and Use Committee at the University of California, Davis, CA 95616. Consent of the animal owner was obtained prior to use of the animals for this research.

## 3. Results 

Mature cows had very high* A. marginale* seropositive AP and TP, from 89.55% at baseline to 95.49% on the final sample days (Tables 2 and 3). By the end of the follow-up period, heifers had also attained a high* A. marginale* carrier status, with TP of 68.68% and 92.38% for group 1 and 2 heifers, respectively. The* A. marginale *seroprevalence was significantly different between each of the study groups at their first sampling day (*P* = 0.045 for group 2 heifers versus group 1 heifers and *P* < 0.0005 for all other comparisons). Over the course of the study, all age groups had an increase in* A. marginale* seroprevalence but this was only statistically significant for the heifer groups (*P* < 0.0005). At the end of the follow-up period,* A. marginale* seroprevalence was no longer significantly different between group 2 heifers and the cow group (*P* = 0.958). The seroprevalence of group 2 heifers continued to be higher than the group 1 heifers at study completion (*P* = 0.003). The* A. marginale* seroprevalence (68.68%) of group 1 heifers at the end of the follow-up period was significantly greater than the seroprevalence (41.13%) of group 2 heifers at study onset (*P* = 0.014).

The pasture location, number of animals, and days at risk per pasture as well as incident cases can be found in Table 4. Incidence density rates and their 95% CIs for different groupings of study animals and pasture exposures were calculated using the observed cases (not estimates based on TP calculations) and are presented in Table 5. Group 2 heifers exposed to pasture B had the highest IDR (2.44 cases/1000 heifer-days). The IRR for* A. marginale* infection is presented in Table 6. Incidence rate ratio (group 2 heifers exposed to pasture B as denominator) was the lowest for group 2 heifers exposed to pasture A (4.12) and was significantly greater than the cow group (2.00) (*P* = 0.012) but not significantly greater than group 1 heifers (3.06) (*P* = 0.288). Group 1 heifers also had a significantly higher IRR (3.06) than the cow group (2.00) (*P* = 0.048). No other significant differences were found.

There were significant differences in IDR for animals as seasonal or pasture exposure changed. Group 2 heifers had a significant decrease in infection rate (*P* = 0.012) when they were moved from annual rangeland pasture (March 1, 2013, to May 10, 2013: IDR = 10.04 cases/1000 heifer-days) to valley irrigated pasture (May 10, 2013, Sep. 10, 2013: IDR = 2.44 cases/1000 heifer-days). In contrast to group 2 heifers, during the time period from May 17, 2013, to Aug. 5, 2013, group 1 heifers, on irrigated mountain meadow pasture, had a high infection rate (IDR = 7.26 cases/1000 heifer-days).

No ticks were found on any of the study cattle during the course of the study. No illnesses were reported in any study cattle that corresponded with clinical signs of bovine anaplasmosis. The herd manager regularly observed and treated infectious bovine keratoconjunctivitis but the exact number of treatments was not recorded.

## 4. Discussion

Cattle in this herd appear to have become* A. marginale* carriers and hence adequately protected from clinical bovine erythrocytic anaplasmosis. Previous literature has suggested that herds with seroprevalence between 1% and 40% would be considered susceptible to new infections from within the herd or from outside the herd via introduction of infected carriers (wildlife reservoirs, introduced cattle) or contaminated fomites (veterinary equipment) [7]. Using 60% seroprevalence against* A. marginale* as a benchmark, the study population well exceeded this threshold.

It is expected that naive animals over a year of age that are infected with* A. marginale* would present with clinical signs. However, there were no observed clinical cases during the course of this study. Several variables may play a role in this including strain and pathogenicity variation as well as animal factors such as health at the time of infection and variation in immune response by this specific breed of livestock. Specifically, one would have expected to see clinical disease in the mature cow that seroconverted during the study. Although it is possible she was infected for the first time during the study, it is also possible that she was a false negative during the initial sampling period.

The difference in IDR across cohorts during similar seasonal periods may be explained by a difference in seasonal vector abundance or feeding activity in various pasture conditions. The high infection rate observed for group 2 pasture A corresponds to a peak of* Dermacentor occidentalis *activity observed at Sierra Foothill Range Field Station in Yuba County, CA, which peaked in March and April in two consecutive years (1987 and 1988) and was largely over by May of those years [21]. The Sierra Foothill Range Field Station is located at 200 m elevation and has similar climate to both of the group 2 heifer pasture exposures. Tick activity decreases dramatically as ambient temperatures increase [21]. The increased elevation of the mountain meadow pasture may have resulted in cooler temperatures in spring and early summer and may have accounted for continued tick activity after group 1 heifers were moved there in May. Another possible contributing factor is that tick vectors in the mountain meadow may have overwintered and passed through early spring with a scarcity of large mammalian hosts but rapidly fed on the cattle herd when the herd was confined to the mountain meadow pasture. Tick vector scenarios are speculative since no ticks were found on cattle to confirm their vector role for* A. marginale* in this study. Mechanical vectors of* A. marginale* infection such as biting flies, vaccinations, and other iatrogenic exposures may also be seasonal and create the results observed in this study.

Additionally, although the midpoint of the age range for group 1 heifers was approximately 5 months younger than the group 2 heifers, the group 1 heifer seroprevalence after 80 days was significantly higher than what was measured for group 2 heifers on day 0. This indicates that in just 80 days group 1 heifers were able to make up for the 5-month age difference (lifetime vector exposure) and cross the benchmark of 60%* A. marginale* seroprevalence, suggesting that pasture exposure at specific critical times is more important than overall exposure time. Further studies are warranted to identify the level of tick activity and the correlation with* A. marginale* infection incidence for various pastures to assist ranch managers in herd health decision-making processes related to anaplasmosis prevention.

Ticks are the most efficient vector of* A. marginale* transmission [22–24] and are suspected to be the vector in this study despite the inability to collect any during cattle inspection at time of blood sample collection. However, animal temperament, dark colored hides, restraint in a squeeze chute, and cursory examinations may have limited the ability to collect ticks during examination. It is also possible that ticks were present on cattle earlier and were no longer feeding by the time of animal processing and sample collection.

*Dermacentor *spp. are the most important tick vectors of* A. marginale* in the United States [3]. Of this genus,* D. occidentalis* is known to feed on black-tailed deer (*Odocoileus hemionus columbianus*), mule deer (*O. hemionus hemionus*), and cattle [25]. Host predilection is important given the susceptibility of black-tailed deer to* A. marginale* which may make black-tailed deer the most important wildlife reservoir of* A. marginale* in California and hence the potential source of infections in this study [25]. However, mechanical transmission of* A. marginale* is well documented and biting flies are known vectors of* A. marginale* [5, 11–13] as are veterinarians and husbandry personnel performing procedures such as vaccination or identification using ear tag application or tattooing which may transfer blood from an infected to susceptible animal [3, 10]. Cattle in this study shared vaccine injection needles and ear tag pliers during processing times which is also a potential route of infection.

The IDR for infection measured in the two heifer groups appeared to be adequate to ensure herd seroprevalence of 60% by the time the study cattle were 2 years old. The lower range of the 95% CI for the IDR calculated for heifers during the high infection rate periods was 4.30 cases per 1000 heifer-days (Table 5). Assuming that tick activity is responsible for the infection rates observed in this study and noting that high tick activity was measured over a three-month period in California beef cattle pastures [21], a 77.4%* A. marginale* seroprevalence would be expected without any other exposure during the remaining 9 months of the year:

(2) 4.30  cases1000  heifer-days×100  heifers×90  daysyear×2  years=77.40  cases100  heifers.

Such an estimate is the low limit for the 95% CI of the IDR and TP values found in this study suggest that a higher* A. marginale* carrier status can be anticipated.

In summary, there was sufficient natural infection of* A. marginale* to prevent an outbreak of bovine erythrocytic anaplasmosis in the study herd; hence, no changes to the herd management protocols to prevent clinical anaplasmosis were warranted. However, cattle ranchers, veterinarians, and livestock advisers should monitor seroprevalence in their herds to ensure that animals continue to become carriers before they become adults to prevent severe disease. The impact of ecosystem change on vector-borne disease is difficult to assess and for this reason continuous monitoring for* A. marginale* infection rates in sentinel herds is recommended to protect California's beef production. Studies with more herds are needed to investigate whether anaplasmosis is a reemerging threat to beef cattle in California before requiring changes to current herd management practices. Additional study of the potential importance for mechanical vectoring as a suitable alternate to biologic exposure is an important future research objective.

## Figures and Tables

**Table 1:** Summary of descriptive characteristics for three cattle cohorts from a beef herd in California which were followed up for *Anaplasma marginale *seroconversion.

Age group	Group 1 heifers	Group 2 heifers	Cows
Number sampled	40	66^*∗*^	37
Age on study day 0	9–12 months	15–17 months	>2 years
Study days sampled	77, 157	0, 70, 193	70, 157
Pastures during study	C (80 days)	A (70 days), B (123 days)	C (80 days)
Study months (2013)	May–August	March–September	May–August

^*∗*^Sixty-six heifers were sampled on day 0. One heifer was lost to follow-up between day 0 and day 70 and a second heifer was lost to follow-up between day 70 and day 193. These heifers tested negative for *A. marginale* every time they were sampled.

**Table 2:** Apparent seroprevalence and 95% CI for *Anaplasma marginale* at baseline and final sampling times and *P* values for McNemar's test comparing seroprevalence within groups at baseline and final sampling times for three cattle cohorts from a beef herd in California which were followed up for *A. marginale* seroconversion.

Age group	Baseline sample		Final sample	
	AP (%)	95% CI	AP (%)	95% CI
Group 1 heifers^*∗*^	22.50^a^	(9.56, 35.44)	67.50^c^	(52.98, 82.02)
Group 2 heifers^*∗*†^	42.42^a^	(30.50, 54.35)	89.06^d^	(81.42, 96.71)
Cows^‡^	86.49^b^	(75.47, 97.50)	91.89^d^	(83.10, 100.00)

^a,b,c,d^Within a column, values with different superscript letters are significantly different (*P* < 0.05).

^*∗*^McNemar's test *P* value < 0.001.

^†^Two group 2 heifers were lost to follow-up between the initial and final sample days.

^‡^McNemar's test *P* value *P* = 0.50.

**Table 3:** True seroprevalence and 95% CI for *Anaplasma marginale* at baseline and final sampling times and *P* values for McNemar's test comparing TP within groups at baseline and final sampling for three cattle cohorts from a beef herd in California which were followed up for *Anaplasma marginale* seroconversion.

Age group	Baseline sample		Final sample	
	TP (%)	95% CI	TP (%)^1^	95% CI
Group 1 heifers^*∗*^	19.23^a^	(6.02, 35.92)	68.68^c^	(51.16, 83.06)
Group 2 heifers^*∗*†^	41.13^b^	(27.69, 54.56)	92.38^d^	(80.99, 100.00)
Cows^‡^	89.55^c^	(73.07, 99.57)	95.49^d^	(80.17, 100.00)

^a,b,c,d^Within a column, values with different superscript letters are significantly different (*P* < 0.05).

^*∗*^McNemar's test *P* value < 0.001.

^†^Two group 2 heifers were lost to follow-up between the initial and final sample days.

^‡^McNemar's test *P* value *P* = 0.50.

**Table 4:** Summary of population at risk, incident cases, and time at risk of *Anaplasma marginale* infection for cattle followed up to determine rate of *A. marginale *infection.

Age group	Group 1 heifers	Group 2 heifers^*∗*^		Cows
Pasture	C^§^	A^†^	B^‡^	C^§^
Population at risk	31	37	10	5
Incident cases	18	26	3	2
Days at risk	80	70	123	80

^*∗*^Group 2 heifers were grazed on pasture A for the first 70 study days and on pasture B for the remaining 123 study days.

^†^Annual rangeland pasture, approximately 160 m elevation, Tehama County, CA.

^‡^Valley irrigated pasture, approximately 120 m elevation, Tehama County, CA.

^§^Mountain meadow irrigated pasture, approximately 1060 m elevation, Plumas County, CA.

**Table 5:** Summary of incidence density rate (IDR) per 1000 cow-days for three cohorts of California beef cattle followed up for *A. marginale* infection by age group/pasture exposure, age group, and stage of maturity.

Age group	IDR	95% CI
Group 1 heifers, pasture C^*∗*^	7.26	(4.30, 11.47)
Group 2 heifers, pasture A^†^	10.04	(6.56, 14.76)
Group 2 heifers, pasture B^‡^	2.44	(0.50, 7.12)
Group 2 heifers, pasture A + pasture B^§^	9.29	(5.58, 12.00)
Combined heifer groups^||^	8.39	(6.16, 11.18)
Group 2 heifers, pasture A and group 1 heifers^*∗*^	8.68	(5.81, 12.50)
Cows, pasture C^*∗*^	5.00	(0.61, 18.05)
Combined heifers and cows^¶^	8.17	(6.04, 10.81)

^*∗*^Cows and group 1 heifers were maintained on pasture C throughout their study period.

^†^Group 2 heifers were maintained on pasture A from study day 0 to day 70.

^‡^Group 2 heifers were maintained on pasture B from study day 71 to day 193.

^§^Incident cases and at-risk time combined for all of the group 2 study period.

^||^Incident cases and at-risk time combined for all of groups 1 and 2 study period.

^¶^Incident cases and at-risk time combined for all study animals throughout the study period.

**Table 6:** Summary of incidence rate ratio (IRR) for three cohorts of California beef cattle followed up for *A. marginale* infection by age group/pasture exposure, age group, and stage of maturity.

Age group	IRR (*P* value)
	Age group/pasture
Group 1 heifers, pasture C^*∗*^	3.06 (0.048)^a^
Group 2 heifers, pasture A^†^	4.12 (0.012)^a^
Group 2 heifers, pasture B^‡^	Reference
Cows, pasture C^*∗*^	2.00 (0.052)

^*∗*^Cows and group 1 heifers were maintained on pasture C throughout their study period.

^†^Group 2 heifers were maintained on pasture A from study day 0 to day 70.

^‡^Group 2 heifers were maintained on pasture B from study day 71 to day 193.

^a^Relative risk significantly greater for these groups at the 95% confidence level.
